# Adsorption Removal of 17β-Estradiol from Water by Rice Straw-Derived Biochar with Special Attention to Pyrolysis Temperature and Background Chemistry

**DOI:** 10.3390/ijerph14101213

**Published:** 2017-10-11

**Authors:** Xiaohua Wang, Ni Liu, Yunguo Liu, Luhua Jiang, Guangming Zeng, Xiaofei Tan, Shaobo Liu, Zhihong Yin, Sirong Tian, Jiang Li

**Affiliations:** 1 College of Environmental Science and Engineering, Hunan University, Changsha 410082, China; Wangxiaohua6999@126.com (X.W.); jiangluhua@hnu.edu.cn (L.J.); zgming@hnu.edu.cn (G.Z.); tanxf@hnu.edu.cn (X.T.); yinzhihong@hnu.edu.cn (Z.Y.); tsirong@hnu.edu.cn (S.T.); lijiang1304@163.com (J.L.); 2 Key Laboratory of Environmental Biology and Pollution Control, Hunan University, Ministry of Education, Changsha 410082, China; 3 School of Architecture and Urban Planning Hunan City University, Yiyang 413099, China; 4 School of Architecture and Art, Central South University, Changsha 410082, China; liushaobo23@aliyun.com; 5 School of Metallurgy and Environment, Central South University, Changsha 410083, China

**Keywords:** endocrine disrupting chemicals, 17β-estradiol, rice straw biochar, pyrolysis temperature, adsorption

## Abstract

Rice straw biochar that produced at three pyrolysis temperatures (400, 500 and 600 °C) were used to investigate the adsorption properties of 17β-estradiol (E2). The biochar samples were characterized by scanning electron microscopy (SEM), Fourier-transform infrared spectroscopy (FTIR), elemental analysis and BET surface area measurements. The influences of pyrolysis temperature, E2 concentration, pH, ionic strength, background electrolyte and humic acid were studied. Kinetic and isotherm results illustrated that the adsorption process could be well described by pseudo-second-order and Freundlich models. Experimental results showed that ionic strength had less influence on the adsorption of E2 by 500 and 600 °C rice straw biochar. Further, multivalent ions had positive impact on E2 removal than monovalent ions and the influence of the pyrolysis temperature was unremarkable when background electrolyte existed in solutions. The adsorption capacity of E2 decreased with the pH ranged from 3.0 to 12.0 and the humic acid concentration from 2 to 10 mg L^−1^. Electrostatic attractions and π-π interaction were involved in the adsorption mechanisms. Compared to low-temperature biochar, high-temperature biochar exhibited a better adsorption capacity for E2 in aqueous solution, indicated it had a greater potential for E2 pollution control.

## 1. Introduction

As early as in 1993, the paper “Estrogen as an Environment Pollutant” had suggested that hormones excreted into the environment by humans and animals were present in sufficient quantities to disrupt the environment [1]. Recently, the related research about the endocrine disrupting compounds (EDCs, i.e., environmental hormone) pollution has caused the extensive concern. EDCs is a kind of chemicals that may disrupt the endocrine systems of animals and humans through mimicking natural hormones, inhibiting the action of hormones, or alter the normal regulatory function of the immune nervous and endocrine systems [2]. As a common of EDCs, 17β-estradiol (E2) was detected in water environment, including surface water, sea water, tap water, and wastewater in recent years around the world [3,4,5,6]. It was reported that E2 can disrupt endocrine system even at ng L^−1^ scale [7] and its disrupting effect is 1000–10,000 times greater than that of nonylphenol [8]. Through accumulating in food chain, it may induce adverse effects on human and animals, such as, anomalous growth, problem of reproductive health, low birth rate and high tumor rate [9]. Therefore, it is of great significant to explore some feasible and effective way to control the E2 pollution.

Among the conventional and advanced treatment methods for removal of E2 from water, such as, physical means, biodegradation, and chemical technologies [10], adsorption has brought extensive attention because of its obvious advantages as easy access, simple and environmental-friendly technique, good removal effects and based on waste control by waste. Various adsorbents have been used to removal E2 from water based on the previous reported literatures. For instance, single-walled or multi-walled, carbon nanotubes, granular activated carbon (GAC), chitin, chitosan, ion exchange resin and graphene nanomaterials have been applied to eliminate E2 from aqueous solution by adsorption [11,12,13,14,15,16]. However, the resource constraints or complicated production process and/or limited adsorption capacity will restrict the practical application of these adsorbents. Therefore, low-cost and easy fabrication materials are called attention to the removal of E2 from aqueous solutions.

Biochar, as a carbon-residue derived from the thermal conversion of waste biomass under limited oxygen or anaerobic conditions [17], has been widely used in water environmental improvement as a result of its wide availability of feedstock, low-cost, favorable physical/chemical surface characteristics and high adsorption-efficiency [18,19]. The use of biochar for effective removal of organic and inorganic contaminants from water has been demonstrated by numerous researches. For example, Sun et al. noted that biochar prepared from anaerobic digestion residue, palm bark and eucalyptus can effectively remove cationic methylene blue dye [20]. Similarly, Sewu et al. reported the highly efficient adsorption of cationic dye by biochar derived from Korean cabbage waste [21]. Taha et al. demonstrated that biochar had higher adsorptive capacity of fifteen different pesticides from water than charcoal [22]. Meanwhile, many studies also showed that biochar exhibited the removal of heavy metal in aqueous solution [23,24]. It is noteworthy that several works have paid attention to adsorption of E2 by biochar. For instance, Sangeeta et al. used bone char derived from waste cattle bones to remove E2 from aqueous solutions and the results showed that the adsorption efficiency reached to 95.3% with a bone char dosage of 50 g L^−1^ [25]. Li et al. focused on investigating the adsorption characteristics of E2 by rice straw biochar [26]. The mechanisms responsible for E2 adsorption by biochar can be ascribed to the chemical composition and characteristics of biochar. It highly relied on the nature of raw material and the pyrolysis parameters. Among pyrolysis parameters, temperature greatly affects the physiochemical characteristics of biochar (i.e., the porosity, aromaticity, surface area and cation exchange capacity). It is a crucial factor for the pyrolysis process of raw material. Nevertheless, few researches were employed to establish the connection between E2 adsorption and biochar characteristics on the basis of different pyrolysis temperature. Therefore, it is imperative to explore the underlying mechanisms on E2 adsorption by biochars derived from different temperatures. Meanwhile, probing the related influencing factors for adsorption of E2 and finding a high quality and inexpensive adsorbent of biochar is necessary.

Rice straw is an agricultural by-product, the dry stalks of cereal plants, after the grain and chaff have been removed. Straw makes up about half of the yield of rice. When rice straw is burned or ploughed under, it may lead to air pollution or generate leachates. In this study, rice straw was prepared as biochar at different temperatures (400, 500 and 600 °C) to explore its adsorption behaviors of E2. Besides, related influence factors such as E2 concentration, contact time, pH, ionic strength, background electrolyte and humic acid on the removal of E2 from aqueous solutions were also investigated systematically. Finally, the adsorption mechanism of E2 by rice straw biochar was also discussed.

## 2. Materials and Methods

### 2.1. Chemicals

E2 (molecular weight 228.29, 98% in purity) was purchased from Sigma–Aldrich Corporation (St. Louis, MO, USA). A stock solution (2.5 mg mL^−1^) of E2 was prepared by dissolved in the analytically pure methanol. All chemicals including NaCl, KCl, CaCl_2_, MgCl_2_, NaNO_3_, Na_2_SO_4_ and Na_3_PO_4_ were analytical reagent grades and obtained from Shanghai Chemical Corp. Experimental water is high-purity water (18.25 MΩ cm^−1^) produced by Millipore Milli-Q water purification system (Millipore, Bedford, MA, USA).

### 2.2. Biochar Preparation

The rice straws were collected from the farms in Yiyang, Hunan Province, China. After washed with water, the samples were dried in the sun, and then smashed it to pass through a 100 mesh sieve (0.147 mm). Then, a lab-scale tubular reactor (SK-G08123K, Tianjin Zhonghuan Experimental Furnace Co. Ltd., Tianjin, China) was used to pyrolyze rice straws in nitrogen flow (50 mL min^−1^) with a heating rate of 5 °C min^−1^ and hold for 2 h at 400, 500 and 600 °C. After the reactor was cooled, the resulted biochar samples (BCs) were taken out and then stored in airtight desiccators before use. According to the pyrolysis temperature, the prepared biochar samples were referred as RS400, RS500 and RS600, respectively.

### 2.3. Biochar Characterization

An elemental analyzer (Vario EL III, Elementar, Analyser systeme GmbH, Hanau, Germany) was used to calculate the elemental compositions of BCs. N_2_ adsorption and desorption measurements (Micromeritics TriStar II 3020, Micromeritics Corporate, Norcross, GA, USA) was applied to characterize the BET surface area and pore structure of samples. The morphologies research on samples by SEM (JSM-7001F, JEOL, Tokyo, Japan). FT-IR spectra of BCs were carried out by a spectrophotometer (Nicolet 6700, Thermo Nicolet Co., Waltham, MA, USA) and the range of the scanning wave numbers were 500–4000 cm^−1^. For the analysis of zeta potentials, 0.01 g BCs were added to 50 mL Milli-Q water and the pH (ranged from 3.0 to 12.0) was adjusted by diluting 0.1 M HCl or NaOH aqueous solutions. Measurement of zeta potential of sorbents was conducted by a zeta potential instrument (Zetasizer, nano-ZS90, Malvern Instruments, Malvern, UK). 

### 2.4. Adsorption Experiments

Desired E2 concentrations used in batch experiments were prepared by appropriately diluting the stock solutions with deionized water and successive dilutions 0.01 g BCs were added into 50 mL E2 solution with a fixed concentration. All the adsorption experiments were performed in conical flasks on a thermostatic water bath oscillator and shaken at 28 °C and 160 rpm. After oscillation, all solutions were filtered through 0.45 μm membrane filters.

An F-4600 fluorescence spectrophotometer (Hitachi, Tokyo, Japan) was used to investigate the E2 concentrations of filtrates. The excitation source is 450 W xenon lamp and the photomultiplier Tube voltage was set at 700 V. Within a set emission range from 290 to 400 nm and the wavelength of excitation 280 nm, the excitation and emission matrix spectra were collected at 5 nm. The speed of scan is 12,000 nm min^−1^. The fluorescence intensity of E2 was collected at Ex/Em = 280 nm/310 nm [27]. The amount of E2 adsorbed onto BCs was obtained by simple calculation formula of differential concentration. The E2 adsorption capacity (*q_e_*) and the adsorption efficient (*E*, *%*) were calculated as follows:

(1) $$q_{e}=\frac{\left(C_{O}-C_{e}\right)\times V}{M}$$

(2) $$E\left(\%\right)=\frac{\left(C_{O}-C_{e}\right)}{C_{o}}\times 100\%$$

where *C_o_* and *C_e_* are the initial and equilibrium concentrations of E2 (mg L^−1^), respectively; *V* is the volume of adsorption solution (L); *M* is the weight of biochar (g).

The impact of initial concentration on the adsorption efficiency was tested by adding 0.01 g BCs (RS400, RS500 and RS600) to different concentrations of solutions (0.2–6 mg L^−1^) with shaking 24 h.

Adsorption kinetic studies were conducted by adding 0.01 g BCs (RS400, RS500 and RS600) to 50 mL of the 6 mg L^−1^ E2 solutions, and then the sample was shaken for the preset time periods (5–1440 min). 

The impact of initial solution pH and ionic strength on E2 adsorption were conducted by adding 0.01 g BCs (RS400, RS500 and RS600) into 1 mg L^−1^ E2 solutions (50 mL). The pH value was adjusted by NaOH or HCl solution (0.1 M) ranged from 3.0 to 12.0. The ionic strength was adjusted through adding NaCl (0.1, 0.01, 0.001 M) into 1 mg L^−1^ E2 solutions (50 mL).

The effects of background electrolyte ions and humic acid on the adsorption capacity of BCs for E2 were investigated by adding 0.01 g BCs (RS400, RS500 and RS600) into 6 mg L^−1^ E2 solutions (50 mL). The influences of background electrolyte ions were examined by adding 0.01 M NaCl, KCl, CaCl_2_, MgCl_2_, NaNO_3_, Na_2_SO_4_ and Na_3_PO_4_, respectively, into the E2 solutions. In the study of the influence of humic acid, the concentration of humic acid follows this sequence: 0, 2, 4, 6, 8 and 10 mg L^−1^, respectively.

## 3. Results and Discussion

### 3.1. Characterization of Biochar

#### 3.1.1. SEM and BET Analysis

SEM provides a more direct view of the surface structure change of BCs. According to the SEM images (Figure S1), the morphology changes as pyrolysis temperature increased. Under the same magnifications (five thousand times), it can be seen that BCs presents tube bundles structure. With the rise of pyrolysis temperature, the decomposition of BCs improved and it clearly observed in Figure S1c. This phenomenon indicated that the structure of biochar was well carbonized at high pyrolysis temperature due to the removal of volatile matter such as cellulose and hemicellulose [28]. The nitrogen adsorption–desorption isotherms of BCs are shown in Figure S2. According to IUPAC (1985), it belongs to the Type II isotherms, which indicates that (1) a typical case of multilayer adsorption happened on porous media; (2) a stronger interactions happened between adsorbent and adsorbate; and (3) a smaller slope represents the form of multilayer disperse when p/p_0_ from 0.3 to 0.8. As displayed in Figure S2, the slope of RS600 is obviously smaller than RS400 and RS500 and the pores of BCs are mainly mesopores (pores of widths between 2 nm and 50 nm).

The BET surface area and the pore diameter of BCs that produced at different temperatures (400, 500 and 600 °C) were presented in Table S1. When the pyrolysis temperature increased from 400 °C, 500 °C to 600 °C, the surface area of BCs showed a rise first followed by a decline and RS500 with the largest surface area values (7.66 m^2^ g^−1^). It reflected that there is more contacting area than the other two samples. The number of micropores significantly increased with the removal of volatile matter, giving rise to an increase in surface area. Above 500 °C, structural ordering, pore widening and/or the coalescence of neighboring pores seem to predominate, leading to a decrease in surface area. Besides, as a result of softening, melting, fusing and carbonization, pores in the biochars might be partially blocked [29]. Instead, the pore size and pore diameter decreased at first, then raised. RS600 had the largest pore size, pore diameter and pore volume, it might be ascribed to the sample expansion and cleavage with temperature rise. The pore volume values were between 0.0158 cm^3^ g^−1^ and 0.0175 cm^3^ g^−1^ with small differences, which indicated that pyrolysis temperatures have no major effect on pore volume. The data and figure of pore size indicated that mesoporous (between 2 nm and 50 nm) were the main morphology of BCs, which is consistent with the measured value of pore size in the above study. 

#### 3.1.2. Elemental Analysis

Elemental composition of BCs produced at three different pyrolysis temperatures are listed in Table 1. As seen, BCs were carbon (C) rich samples with carbon contents around 52–59%. When the temperature rose from 400 to 500 °C, C and nitrogen (N) contents increased from 52% to 59%, and 0.72% to 0.78%, respectively. The high C content in RS500 suggest that the biochar carbonization can be accelerated as the pyrolysis temperature increases [30]. The increase of N was recorded in higher pyrolysis temperature, it may be related to recalcitrant N occurring in heterocyclic compounds [31]. However, with a temperature of 600 °C, the contents of C and N decreased to 56% and 0.42%, respectively. As is known, the changes in C content occurred concurrently with the hydrogen (H) and oxygen losses [32]. The loss of N can be ascribed to the emission of ammonia and other volatile organic compounds containing nitrogen during pyrolysis process [33,34,35]. The sulfur (S) content of BCs showed a similar pattern as carbon and nitrogen, i.e., it increased at first and then decreased, but it changes slightly. This indicated that the influence of pyrolysis temperature on the S content of BCs was not obvious. Biochar from paved-feedlot manure and turkey litter had the same change trend in such elements content [36]. On the other hand, the H contents dropped with the rise of temperature and the result was consistent with previous studies [37,38]. Losses in H at high pyrolysis temperature may be ascribed to the breaking of weaker bonds within biochar structure [39]. As an important indicator of the release of inorganic from organic matter, the C/N ratio of BCs was positively correlated with increasing temperature. This suggested that the higher the pyrolysis temperature, the more inorganic N of BCs could be release.

#### 3.1.3. FTIR Analysis

**Figure 1:**
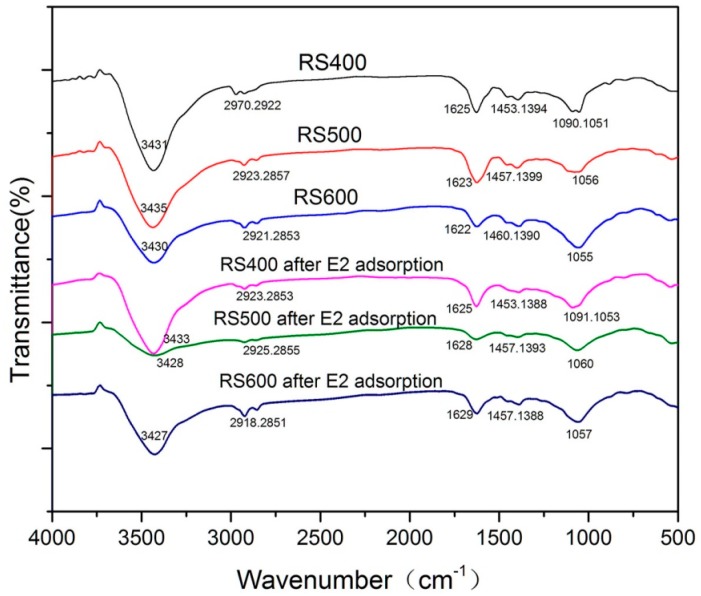
FTIR spectra of BCs and BCs after E2 adsorption.

FTIR is one of the commonly used methods for chemical structure analysis and identification. The FTIR spectra of the BCs before adsorption are showed in Figure 1. The peaks were mainly appeared at about 3427–3435 cm^−1^, 2851–2970 cm^−1^, 1622–1628 cm^−1^, 1388–1460 cm^−1^, 1050–1091 cm^−1^, respectively. The major broad peaks of BCs around 3430 cm^−1^ revealed the presence of O-H groups in the celluloses and hemicelluloses components of BCs [40,41,42]. Vibration band at 2970 cm^−1^ could be attributed to asymmetric -CH_3_ stretching vibration [43]. Peaks at about 2920 cm^−1^ and 2850 cm^−1^ indicated the presence of asymmetric and symmetric stretching vibrations of -CH_2_- [44]. The peak at about 1622 cm^−1^ was an evidence for C=C stretching in the aromatic ring, and it suggested the presence of lignin constituents [38]. Peak at 1456 cm^−1^ might be ascribed to C-H in plane bending vibrations [45]. The peak at 1390 cm^−1^ corresponded to the amine I, which was the characteristic of functional groups C=O, stretching vibration of proteins [46]. The peaks appear near at 1399 cm^−1^, 1064 cm^−1^, 1090 cm^−1^ and 1051 cm^−1^ are due to the stretching of the C-O bonds [47,48,49,50]. All the adsorption peaks showed that O-H, -CH_3_, -CH_2_-, C=C, C-H, C=O and C-O were the main functional groups of samples. With the increase of pyrolysis temperature, some peaks had changed. The asymmetric -CH_3_ and -CH_2_- stretching vibration (2970 cm^−1^ and 2922 cm^−1^) of RS400 shifted to asymmetric -CH_2_- and symmetric -CH_2_- (2920 cm^−1^ and 2850 cm^−1^), respectively. The C-O bonds (1090 cm^−1^) disappeared. Thus, the surface functional groups of biochar could be influenced by pyrolysis temperature and the change of biochar properties would have some impacts on the adsorption of E2. At the same time, the FTIR spectra of BCs after E2 adsorption also showed in Figure 1 and the comparative analysis were discussed later.

### 3.2. Adsorption Kinetics

To examine the mechanism of adsorption and potential rate-controlling steps (e.g., chemical reaction and diffusion control), different kinetic models were employed to simulate the experimental data. The typical kinetics models are generally expressed as follows [51]:

The pseudo-first-order model is given as:

(3) $$\ln(q_{e}-q_{t})=\ln q_{e}-k_{1}t$$

The pseudo-second-order model can be presented as:

(4) $$\frac{t}{q_{t}}=\frac{1}{k_{2}q_{e}^{2}}+\frac{t}{q_{e}}$$

where *q_e_* and *q_t_* (mg g^−1^) are the adsorption amount at equilibrium and time *t*, respectively; *k*_1_ (min^−1^) and *k*_2_ (g mg^−1^ min^−1^) are the corresponding adsorption rate constant, respectively.

The Elovich equation is given as [52]:

(5) $$q_{t}=\frac{1}{\beta }\ln\left(\alpha \beta \right)+\frac{1}{\beta }\mathrm{lnt}$$

where *α* (mmol g^−1^ min) is the initial adsorption rate and *β* is related to the extend of surface coverage and the activation energy involved in chemisorption (g mmol^−1^).

The Boyd equation is formulated as [53]:

(6) $$F=1-\frac{6}{\pi^{2}}\overset{\infty }{\underset{m=1}{\sum }}\frac{1}{m^{2}}\exp\left(-m^{2}\mathrm{Bt}\right)$$

where *F* is the fraction attainment of equilibrium at time *t* and it obtained by the expression:

(7) $$F=\frac{q_{t}}{q_{e}}$$

where *q_t_* (mg g^−1^) is the amount of sorbate taken up at time *t* and *q_e_* (mg g^−1^) is the maximum equilibrium uptake and

(8) $$B=\frac{D_{i}\pi^{2}}{r^{2}}$$

where *B* is the time constant (min^−1^), *D_i_* is the effective diffusion coefficient of the E2 ions in the sorbent phase (cm^2^ min^−1^), *r* is the radius of the sorbent particle (cm), assumed to be spherical, and m is an integer that defines the infinite series solution, *Bt* is given as:

(9) Bt=−0.4977−ln(1−F)

Thus, the value of *Bt* can be computed for each value of *F*, and then plotted against time to configure the so-called Boyd plots.

**Figure 2:**
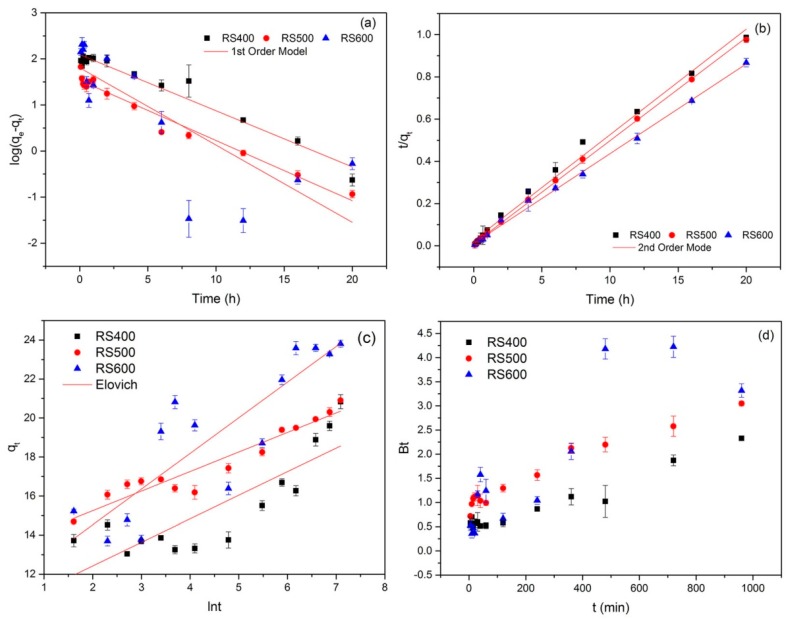
Kinetic plot of the adsorption of BCs on E2. (**a**) pseudo-first-order; (**b**) pseudo-second-order; (**c**) Elovich; (**d**) Boyd (initial concentration = 6 mg L^−1^; biochar dose = 0.01 g; temperature = 301 K).

The kinetic experiments results of different models for E2 adsorbed onto the BCs were presented in Figure 2 and the simulated parameters were listed in Table 2. As shown, the determination coefficient (*R^2^*) of pseudo-second-order model (0.990, 0.998 and 0.997, respectively) for the linear plots were all higher than 0.99 and it was better than pseudo-first-order model (0.955, 0.967 and 0.605, respectively) and the Elovich model (0.691, 0.914, 0.736, respectively). This suggested that the pseudo-second-order kinetics could more accurately describe the adsorption process of biochar on E2. Generally, the pseudo-second-order kinetics model was based on the assumption that the rate-limiting step related to chemisorption [54]. Accordingly, the chemisorption of E2 was the rate-determining step of adsorption process, which involved the chemical interaction between E2 and BCs, such as π-π interaction and hydrogen bonding [15].

Boyd model is usually employed to distinguish sorption controlled between film diffusion and particle diffusion [55]. A straight line passing through the origin is indicative of sorption processed governed by particle-diffusion mechanisms, otherwise they are governed by film diffusion. [56]. Figure 2d shows the linearity plot of *Bt* vs. *t* plots. However, the plots were neither linear nor passed through origin at different pyrolysis temperature, indicating the film diffusion—controlled mechanism.

### 3.3. Adsorption Isotherms

Three different isotherm models, Langmuir, Freundlich, Temkin were carried out to fit adsorption isotherm data and explore the adsorption mechanism of E2 onto biochar surfaces. The equation of Langmuir and Freundlich are two widespread used isotherms for the description of adsorption equilibrium. The Langmuir isotherm based on three assumptions, namely adsorption is limited to monolayer coverage, all surface sites are alike and only can accommodate one adsorbed atom and the ability of a molecule to be adsorbed on a given site is independent of its neighboring sites occupancy [57]. The Freundlich isotherm model is based on the assumption of adsorption on heterogeneous surfaces and allows also treating multilayer adsorption [58]. Temkin isotherm model is also used for representing the equilibrium adsorptive behavior between two phases composing the adsorption system. It is assumed that the heat of adsorption of all the molecules in the layer would decrease linearly with coverage due to adsorbent–adsorbate interactions. The adsorption is characterized by a uniform distribution of binding energies, up to certain a maximum binding energy [59]. These models have generally been used in the form as follows (10)–(12) [60,61], respectively:

Langmuir equation:

(10) $$q_{e}=\frac{K_{L}q_{m}C_{e}}{1+K_{L}C_{e}}$$

Freundlich equation:

(11) $$q_{e}=K_{F}C_{e}^{n}$$

Temkin equation:

(12) $$q_{e}=B\ln\left(A\right)+B\ln\left(C_{e}\right)$$

where *q_e_* (mg g^−1^) and *q_m_* (mg g^−1^) are the equilibrium-sorbed concentration and the maximum adsorption capacity of the adsorbent, respectively. *C_e_* (mg L^−1^) is the E2 concentration in the solution at equilibrium, and *K_L_* (L g^−1^) is the Langmuir constant related to binding energy. The *K_F_* ((mg g^−1^) (mg L^−1^)^−n^) parameter is the Freundlich constant related to adsorption capacity, and *n* is an indicator of the adsorption intensity. The *B* = *RT/b*, *b* is the Temkin constant related to the heat of sorption (J mol^−1^); *A* is the Temkin constant (L mg^−1^), *R* the gas constant (8.314 J mol^−1^ K^−1^), and *T* is the absolute temperature (K).

**Figure 3:**
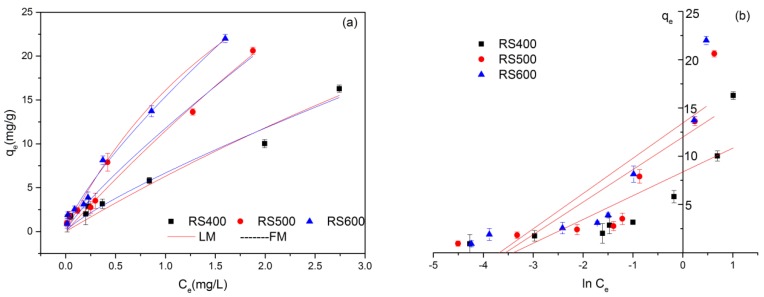
Adsorption isotherms of E2 of biochars produced at three temperatures. (**a**) Langmuir and Freundlich model; (**b**) Temkin model (initial E2 concentration = 0.2–6 mg L^−1^; sorbent dose = 0.01 g; contact time = 24 h).

Figure 3 presents the three isotherms of E2 on RS400, RS500 and RS600 at 28 °C, and the relative parameters calculated from these models were showed in Table 3. It demonstrated that the absorptive ability increased obviously with concentration increasing of E2. Meanwhile, RS500 and RS600 represented better adsorption properties than RS400, but the difference between RS500 and RS600 was not significant. Compared to the correlation coefficient (*R*^2^) values, the values of Freundlich model (*R*^2^ = 0.961, 0.969, 0.947) were slightly higher than Langmuir model (*R*^2^ = 0.945, 0.965, 0.935), and Temkin (*R*^2^ = 0.638, 0.632, 0.682) had the worst imitative effect, which indicated that the adsorption of E2 on biochars fitted Freundlich better than Langmuir and Temkin models. For Freundlich isotherm, *K_f_* values increased with the rise of temperature, suggesting that the adsorption capacity was mounting. The parameter *n* represents the interaction strength between the surface of adsorbent molecule and adsorbent. It could be found that the value of *n* increased at first and then decreased, implying that the process of adsorption was influenced by many factors. The adsorption isotherm of E2 exhibited Freundlich behavior, which indicates that the adsorption happens on a heterogeneous surface by multilayer sorption and it was consistent with the consequence of nitrogen adsorption–desorption isotherms. At an initial concentration of 4 mg L^−1^, all the adsorption capacity of E2 were below the maximum adsorption (*q_m_*) that fitted by Langmuir adsorption equation. Results showed that BCs had best adsorption with the E2 concentration of 6 mg L^−1^. Therefore, 6 mg L^−1^ could be assumed as starting concentration of E2 in experiments.

### 3.4. Effect of Solution pH

**Figure 4:**
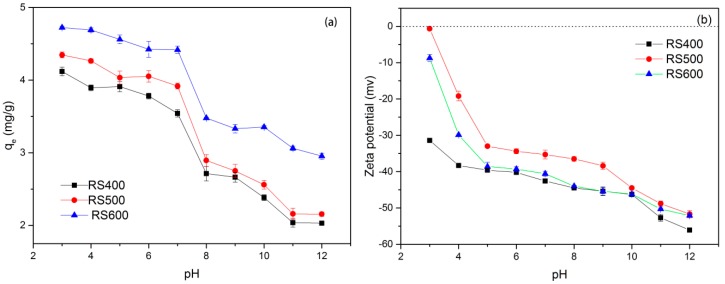
(**a**) Effects of solution pH on E2 by RS400, RS500 and RS600; (**b**) Zeta potential of BCs at different pH. (initial concentration = 1 mg L^−1^; biochar dose = 0.01 g; temperature = 301 K).

Solution pH is a major influencing factor in adsorption process, since it can alter the surface charge of sorbent. Figure 4a illustrates the effect of initial pH on E2 uptake by BCs with pH ranging from 3.0 to 12.0. It could be found that the sorption capacity of E2 relatively stabilized at pH 3–7, decreased rapidly at pH 7–8 and decreased slowly at pH > 8. Evidently, the sorption capacity tended to decrease with the increased value of pH. These phenomena might be ascribed to the change of the surface charge of BCs and the speciation of E2 at different pH values [15]. Moreover, under the same conditions, RS600 and RS500 showed better adsorption efficiency than RS400. It would seem to indicate that the high pyrolysis temperature of BCs would enhance the adsorbability of E2 with pH ranging from 3.0 to 12.0 and this could be attributed to the change of BCs surface functional area. The zeta-potential-pH curves of three biochar are shown in Figure 4b. The zeta potentials of BCs kept negative in the whole pH range and became more negative with increased pH, indicating that the surfaces of biochar particles carried negative charges and the negative charge increased with the increasing of pH. 

### 3.5. Effect of Ionic Strength

**Figure 5:**
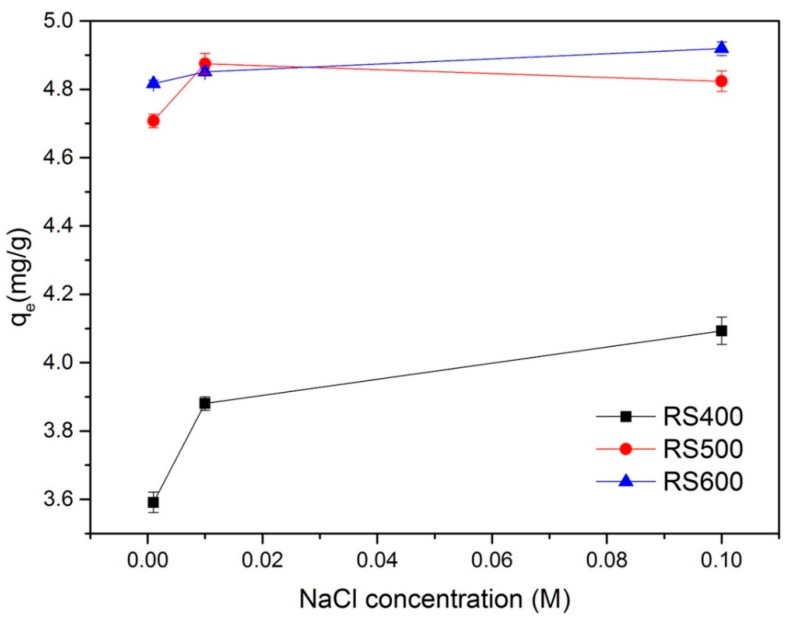
Effect of the ionic strength (initial concentration = 1 mg L^−1^; biochar dose = 0.01 g; temperature = 301 K).

In the process of pollutants adsorption, ionic strength is an important factor affecting the adsorption processes. Thus, a series experiment studies were carried out to understand the effect of ionic strength on the adsorption of E2. As shown in Figure 5, the adsorption of E2 was increased in the presence of NaCl and increased with the increase of its concentration. The phenomenon could be attributed to two potential effects in this process: (1) ionic strength enhanced the activity coefficient of hydrophobic organic compounds and result in decreasing in their solubility (i.e., salting out effect), which was conducive to E2 removal [62]; and (2) the ions might infiltrate into the diffuse double layer over BCs surfaces and reduced the repelling interaction between the adsorbents, resulting in promoting the formation of a more compact aggregation structure (i.e., squeezing-out), which was unfavorable for E2 sorption [63]. The adsorption capacity increased quickly when the NaCl concentration was below 0.01 M, It indicated that the competition of Cl^−^ with E2 for the available binding sites was existed on BCs by a salting out effect and thus enhanced the E2 adsorption. However, when the NaCl concentration was above 0.01 M, the adsorption capacity increased relatively slow, it is suggested that squeezing-out effect may limited the E2 adsorption [64]. Furthermore, the variation tendency of E2 adsorption capacity by BCs in different concentration of NaCl was about the same. It also could be seen that adsorption quantity increased with increasing the pyrolysis temperature, but the difference between RS500 and RS600 was small, which indicated that ionic strength had less influence on the sorption of E2 by BCs that produced at higher temperature. It might be related to the effect of different pyrolysis temperature on surface structure change of BCs.

### 3.6. Effect of Background Electrolyte

**Figure 6:**
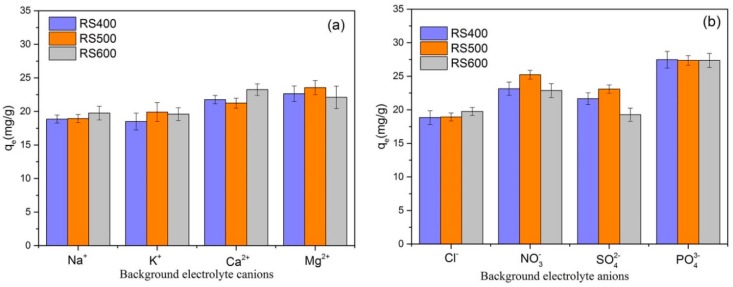
(**a**) Effect of background electrolyte cations on E2; (**b**) Effect of background electrolyte anions on E2 (initial concentration = 6 mg L^−1^; biochar dose = 0.01 g; temperature = 301 K).

The effects of four background cations (Na^+^, K^+^, Mg^2+^ and Ca^2+^ ) and four background anions (Cl^−^, NO_3_^−^, SO_4_^2−^ and PO_4_^3−^) on the E2 adsorption were studied and results were shown in Figure 6. As seen in Figure 6a, the presence of cations slightly improved the E2 removal and different kinds of cations showed different effect on the E2 adsorption. Contrary to the result in previous study [15], the adsorption capacity in the presence of divalent cations (Ca^2+^ and Mg^2+^) higher than monovalent cations (Na^+^ and K^+^). It may be elucidated by the strong interaction between divalent cations with the negatively charged sites on the surface of BCs, which might form more complexes and thus were conducive to remove E2. From Figure 6b, it could be easily found that the effect of trivalent anions (PO_4_^3−^) on adsorption of E2 was better than monovalent and divalent anions (Cl^−^, NO_3_^−^ and SO_4_^2−^). Different background electrolytes had different influences on the E2 adsorption by BCs in differ temperature. In general, the multivalent ions showed better impact on E2 removal than lower ions, except the divalent anions (SO_4_^2−^). It may be ascribed to the high affinity of adsorbent (BCs) with multivalent ions, which enhanced the adsorption efficiency. However, pyrolysis temperature had a negligible effect on E2 removal from solutions and further research is needed into the mechanisms.

### 3.7. Effect of Humic Acid

**Figure 7:**
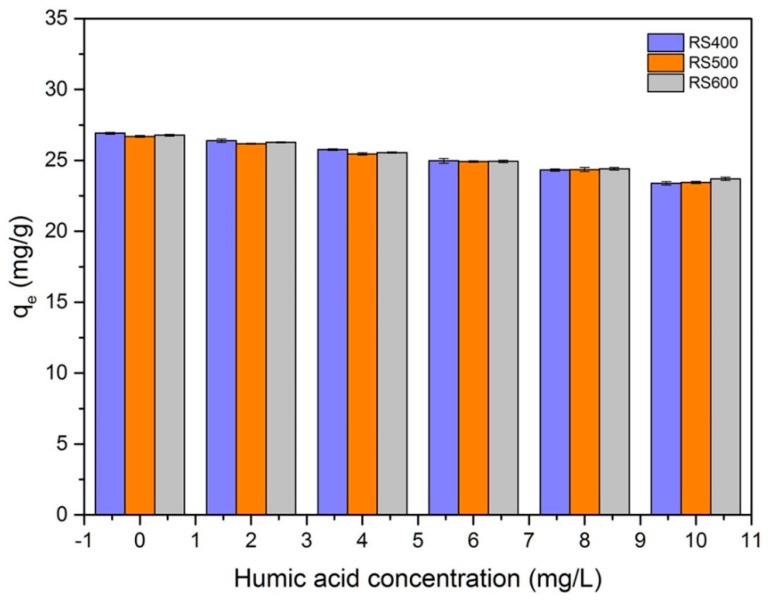
Effect of the humic acid (initial concentration = 6 mg L^−1^; biochar dose = 0.01 g; temperature = 301 K).

It is well known that humic substances are widespread in water and soil, which may influence the removal of pollutants. Figure 7 showed the effects of different concentrations of humic acid on the removal of E2. It can be seen that the adsorption capacity of E2 started to decrease (from 26.91 mg g^−^^1^ to 23.78 mg g^−1^) with the increase of humic acid concentration. These observations could be due to the strong tendency of humic acid to retain hydrophobic organic compounds, which result in degradation of adsorption capacity [14]. It could also be explained by the adsorption of the humic acid containing negatively charged functional groups to the BCs surface, which caused the BCs to be more negatively charged [65,66]. Therefore, the electrostatic repulsion between the negatively charged BCs and humic acid leaded to the decrease of E2 sorption capacity by BCs. As seen, RS400 showed the better adsorption E2capacity than RS500 and RS600. The reasons for this phenomenon could be partially explained by the strong electrostatic repulsion interaction of humic acid with rice straw biochar that produced at high pyrolysis temperature and it may relate to the more developed pore structure of RS500 and RS600. Overall, the addition of humic acid decreased the removal rate to E2.

### 3.8. Possible Mechanisms

The mechanism of adsorption depends on the physical and/or chemical characteristics of the adsorbents as well as on the mass transport process [67]. According to the results of characterization and adsorption isotherm, multilayer adsorption happened on porous and heterogeneous surfaces of BCs. Considering the amount of E2 adsorbed on biochar decreased with the rise of pH values (Figure 4), electrostatic repulsion might play important roles in the adsorption process. Figure 1 compares the FTIR spectra for BCs before and after reaction with E2. As seen, the peak at 2970 cm^−1^ of RS400 became weak after the adsorption, which suggested the loss of hydrocarbon such as C-H. The CH_2_ stretching band centered at 2917 cm^−1^ shifted from asymmetric to symmetric stretching vibrations. The peak at 1090 cm^−1^ for the biochar sample (RS400) was assigned to the C-O bonds, and it disappeared when pyrolysis temperature was increased to 500 and 600 °C. These data indicated that an ignition loss of C-O from BCs with increased temperature. The peaks corresponding to the skeletal vibration of C=C bonds shifted from 1622 cm^−1^ to 1629 cm^−1^ after adsorption of E2, which confirmed that π-π interaction played important roles in the adsorption process between BCs and E2 [68]. Meanwhile, the kinetic research indicated that chemical sorption and film diffusion–controlled might be the happened in the adsorption process. Overall, experiments showed that the adsorption of E2 by BC is a physicochemical process and the mechanism of adsorption included π-π interaction, electrostatic repulsion, film diffusion–controlled and multilayer adsorption between E2 and BCs.

## 4. Conclusions

This study investigated the adsorption of E2 from water using rice straw-derived biochar that produced at three different pyrolysis temperatures. Pyrolysis temperature had great effect on the physiochemical properties of BCs and E2 adsorption capacity. With the pyrolysis temperature increasing, the content of carbon, nitrogen and sulfur increased firstly and then decreased while the hydrogen decreased. Meanwhile, the cleavage of BCs appeared as the temperature increased. Generally, BCs that produced at high pyrolysis temperature exhibited a better adsorption of E2 in batch experiments. In addition, when the pH ranged from 3.0 to 12.0 and the concentration of humic acid increased from 2 to 10 mg L^−1^, the adsorption capacity of E2 decreased from 26.91 mg g^−1^ to 23.78 mg g^−1^. Kinetic data were well simulated by the pseudo-second-order model and film diffusion might be involved in the adsorption process. Equilibrium isotherm data showed a good compliance with the Freundlich model. Ionic strength had less effect on the sorption of E2 by BCs that produced at higher temperature. The E2 removal by BCs involved both chemisorption and physisorption, so is a physicochemical process. As a cost-effective, easily prepared, and environmentally-friendly adsorbent, the high-temperature rice straw biochar could be applied for E2 polluted water treatment.

## Figures and Tables

**Table 1:** Elements analysis of BCs.

Samples	C (%)	N (%)	S (%)	H (%)	C/N
RS400	51.62	0.720	0.224	2.821	71.67
RS500	58.75	0.777	0.246	2.176	75.6
RS600	56.11	0.416	0.219	1.804	135

C: carbon; N: nitrogen; S: sulfur; H: hydrogen.

**Table 2:** Kinetic parameters for adsorption of E2.

Models		RS400	RS500	RS600
Pseudo-first-order	*q_e_* (mg g^−1^)	8.108	4.639	6.090
	*k*_1_ (1 h^−1^)	0.121	0.130	0.167
	*R*^2^	0.955	0.967	0.605
Pseudo-second-order	*q_e_* (mg g^−1^)	20.269	20.693	23.942
	*k*_2_ (g mg^−1^ h^−1^)	0.088	0.215	0.131
	*R*^2^	0.990	0.998	0.997
Elovich	*α*	4853.07	553,319.39	703.32
	*β*	0.829	0.998	0.547
	*R*^2^	0.691	0.914	0.736

**Table 3:** Langmuir and Freundlich isotherm parameters.

Models		RS400	RS500	RS600
Freundlich	*K_f_* (L mg^−1^)	6.858	12.044	13.952
	*n*	0.779	0.812	0.764
	*R*^2^	0.961	0.969	0.947
Langmuir	*q_m_* (mg g^−1^)	59.890	64.888	52.994
	*K_L_* (L mg^−1^)	0.124	0.237	0.374
	*R*^2^	0.945	0.965	0.935
Temkin	*A*	29.877	35.005	39.534
	*B*	2.463	3.370	3.655
	*R*^2^	0.638	0.632	0.682

## Supplementary Materials

The following are available online at www.mdpi.com/1660-4601/14/10/1213/s1. Figure S1: SEM images of BCs produced at various temperatures, Figure S2: N2 adsorption-desorption isotherms of BCs produced at various temperatures, Table S1: The date of BET analysis of BCs.
