# Supplementary material for: Adsorption Removal of 17β-Estradiol from Water by Rice Straw-Derived Biochar with Special Attention to Pyrolysis Temperature and Background Chemistry
Source: Int J Environ Res Public Health. 2017 Oct 11;14(10):1213. doi: 10.3390/ijerph14101213 (PMC5664714; doi:10.3390/ijerph14101213)
Supplement: Supplementary file 1 [file ijerph-14-01213-s001.pdf]

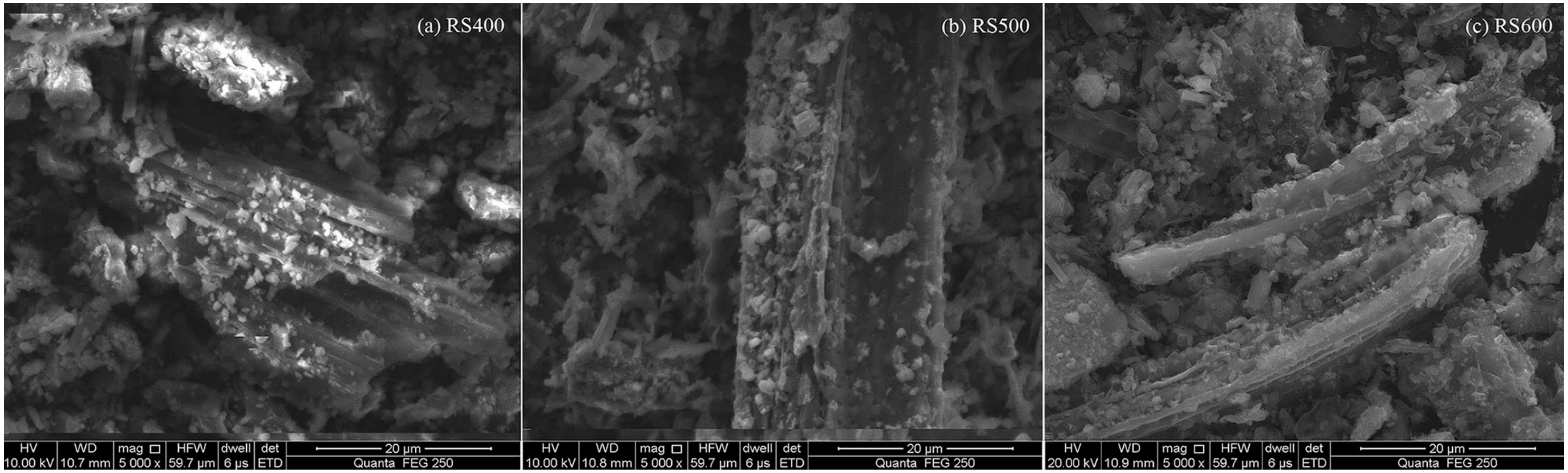


**Figure S1.** SEM images of BCs produced at various temperatures.

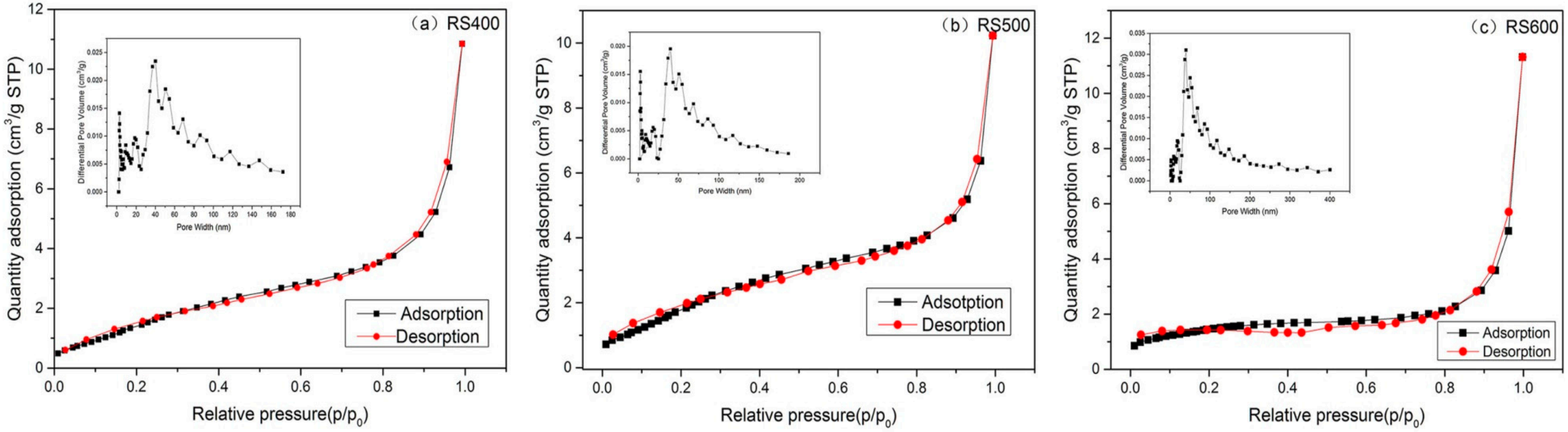


**Figure S2.** N2 adsorption-desorption isotherms of BCs produced at various temperatures.

**Table S1.** The **data** of BET analysis of BCs.

| Sample | Surface Area ($m^2 g^{-1}$) | Pore Size (nm) | Pore Diameter (nm) | Pore Volume ($cm^3 g^{-1}$) |
|---|---|---|---|---|
| RS400 | 5.614 | 11.95 | 10.722 | 0.016776 |
| RS500 | 7.658 | 8.26 | 8.677 | 0.015822 |
| RS600 | 5.137 | 13.63 | 19.552 | 0.017503 |
